# Clinical course of patients on maintenance hemodialysis and COVID-19: a retrospective longitudinal study

**DOI:** 10.7150/ijms.49337

**Published:** 2021-04-12

**Authors:** Guan-nan Jin, Ze-yang Ding, Gan-xun Li, Jun-bo Hu, Ji-hong Liu, Bixiang Zhang, Xiao-ping Chen

**Affiliations:** 1 Department of Nephrology, Union Hosptial, Tongji Medical College, Huazhong University of Science and Technology, Wuhan 430033, Hubei, China.; 2 Department of Surgery, Tongji Hosptial, Tongji Medical College, Huazhong University of Science and Technology, Wuhan 430030, Hubei, China.; 3 Department and Institute of Urology, Tongji Hosptial, Tongji Medical College, Huazhong University of Science and Technology, Wuhan 430030, Hubei, China.; 4 Tongji multidisciplinary Team for Treating COVID-19 (TTTC), Tongji Hosptial, Tongji Medical College, Huazhong University of Science and Technology, Wuhan 430030, Hubei, China.

**Keywords:** maintenance hemodialysis patients, COVID-19, clinical features, outcome, SARS-COV-2

## Abstract

Coronavirus Disease 2019 (COVID-19) emerges as a global pandemic and there is a lack of evidence about the clinical course and outcome of patients on maintenance hemodialysis (MHD). Here we conducted a retrospective longitudinal study aimed to analyze the clinical features and outcome of MHD patients hospitalized with COVID-19. Of 3126 inpatients with COVID-19 at 3 Branches of Wuhan Tongji Hospital from Jan 18th to Mar 9th, 2020, 19 patients were undergoing maintenance hemodialysis. Among the 19 MHD patients with COVID-19, 6 patients (31.6%) died, and 13 patients (68.4%) were able to be discharged. Baseline characteristics, clinical courses, laboratory findings, and dynamic trajectories of major laboratory markers were compared between survivors and nonsurvivors. According to our findings, MHD patients with COVID-19 who experienced non-surviving outcome had more elevated CRP, IL6 and procalcitonin as well as fibrinogen levels at various points compared to survivors. Thus the dysregulation of immune response as well as coagulation abnormalities might be highly involved in the pathological process of COVID-19, contributing to the poor prognosis in MHD patients.

## Introduction

COVID-19 caused by SARS-CoV-2 1 becomes a global pandemic. End-stage renal disease (ESRD) patients on maintenance hemodialysis are specific and highly vulnerable to infections 2-4. To date, detailed clinical characteristics of MHD patients with COVID-19 are still unclear. This retrospective study aimed to analyze the baseline characteristics, clinical courses, laboratory findings, and dynamic trajectories of major laboratory markers of MHD patients with COVID-19, and the differences were compared between survivors and non-survivors.

## Materials and Methods

### Study design, data collection and follow up

This retrospective study enrolled a total of 19 hospitalized MHD patients from 3126 in patients' with laboratory-confirmed COVID-19 infection admitted at 3 Branches of Wuhan Tongji Hospital from Jan 18th to Mar 9th, 2020. During the pandemic, Wuhan Tongji hospital was a designated hospital for the treatment of patients with COVID-19 by the Chinese government, and these 19 patients had been diagnosed as uremia undergoing maintenance hemodialysis before the pandemic and came from different dialysis centers in Wuhan, and were admitted to Tongji hospital after diagnosed as COVID-19 infection. All participants enrolled were laboratory confirmed of SARS-CoV-2 positive by RT-PCR assay of nasal and/or pharyngeal specimens. Patients with a history of renal transplantation or hemodialysis or acute kidney injury were excluded. Survivors were discharged after disappearance of respiratory symptoms for more than three days and negative pharynx swab test of SARS-CoV-2 for twice. Clinical retrospective data was retrieved from the electronic medical records in the Tongji Cloud Hospital Information System (HIS), including demographic features, clinical features, laboratory findings, chest CT images, and treatments. The end date of follow-up was April 26th, 2020, and all patients had definite outcomes (discharged or dead).

### Statistical analysis

Continuous variables were presented as mean with Standard Deviation (SD) or as median with Interquartile Range (IQR). Categorical variables were presented as number (%). We conducted primary analysis using a two-sample t-test or Wilcoxon rank-sum test depending on parametric or nonparametric data for continuous data and Chi-square or Fisher's exact test for categorical data. We compared dynamic changes of laboratory characteristics between survivors and non-survivors during their hospital stay by using a generalized addictive model (GAM). Statistical analysis was performed using SPSS Statistics version 26.0, or R software, version 3.6.2 (R Foundation for Statistical Computing), and two-side P-value <0.05 was considered statistically significant.

## Results

### Baseline characteristics of MHD patients with COVID-19 enrolled in this study

3126 adult patients with COVID-19 were hospitalized in 3 branches of Wuhan Tongji Hospital from Jan 18th to Mar 9th, 2020. After excluding patients with a history of renal transplantation or hemodialysis or acute kidney injury, 19 MHD inpatients (10 male and 9 female) were included in the final analysis. 6 patients died during hospitalization and 13 were discharged. The baseline characteristics were showed in Table 1. The mean age of the patients was 63.1 years [SD 11.8], ranging from 40 and 81. In total, 52.6% patients were over 65 years old. Among these patients, 52.6% were male. The average urea Kt/V was 1.23±0.20. Dry weight average was 64.2 ±3.1 kg. The median dialysis duration was 3yr [IQR 1.0, 3.0]. In total, 6 patients died during their hospital stay with 31.6% mortality rate among 19 MHD patients with COVID-19. The median duration from onset of symptoms to in-hospital death was 21 days [7.0, 28.0]. In survivors, median duration of viral shedding was 20 days [15.0, 35.0]. Coexisting disorders were present in nearly all patients, with hypertension (78.9%) being the most common comorbidity, other comorbidities including cardiovascular disease (31.5%), diabetes (10.5%), COPD (10.5%). Common symptoms on admission were fever (68.4%), cough (68.4%) and short of breath (36.8%). Diarrhea was more common in non-survivors (3/6, 50%). A total of 5 patients were classified as severe COVID-19^5^. Age, gender, Kt/V, dialysis duration and pre-existing comorbidities showed no difference between non-survivors and survivors.

### Laboratory findings of MHD patients with COVID-19 on admission

**Figure 1:**
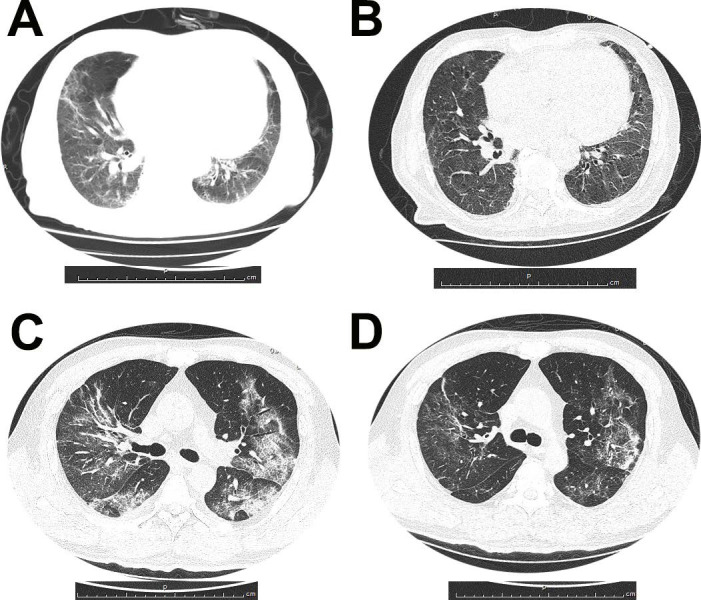
(A and B) Dynamic changes of chest CT images from a 79-year-old man who survived the disease. (A) Bilateral ground-glass opacity and consolidation with interlobular septal thickening on day 10 after symptom onset. (B) After 11 days treatment, chest CT scan from the man showed pulmonary infiltration was absorbed largely. (C and D) Chest CT images from a 53-year-old woman with severe pneumonia, who died for multiorgan failure. (C) Multiple ground-glass opacities and consolidation in both sides of lungs, on day 15 after symptom onset. (D) After 7 days treatment, chest CT image of the woman showed pulmonary infiltration was partially absorbed.

All patients had abnormal chest radiograph and the common abnormities on imaging were ground-glass opacities. Figure 1 demonstrated dynamic CT changes of one survivor and one non-survivor during the clinical course of SARS-CoV-2 infection.

The chest CT images from a 79-year-old man who survived the disease showed the bilateral ground-glass opacity and consolidation with interlobular septal thickening on day 10 after symptom onset. And after 11 days treatment, chest CT scan from the man showed pulmonary infiltration was absorbed largely. Chest CT images from a 53-year-old woman with severe pneumonia, who died for multiorgan failure showed multiple ground-glass opacities and consolidation in both sides of lungs, on day 15 after symptom onset. After 7 days treatment, chest CT image of the woman showed pulmonary infiltration was still existed.

On admission, we observed an average of reduced lymphocyte count, mild anemia, increased cardiac injury markers levels and increased D-dimer level in MHD patients, however there were no differences of these parameters between survivors and non-survivors. Increased fibrinogen occurred in 89.4% (n=17) of MHD patients, importantly, we observed a significant increase of fibrinogen level in non-survivors compared to survivors (6.16 [5.72, 6.62] verses 4.81 [4.18, 5.19], P=0.006), indicating more severe coagulopathy in non-survivors than survivors during SARS-CoV-2 infection.

### Infection-related biomarkers and cytokine profile in MHD patients with COVID-19

Assessment of infection-related biomarkers (CRP, ESR) and cytokine profile (IL-2R, IL-6, IL-8, IL-10, IL-1β, TNF-α, procalcitonin) on admission showed a proinflammatory state in hemodialysis patients with COVID-19 (Table 3). The median level of CRP, ESR, procalcitonin, IL-6, IL-2R, TNF-α levels were clearly elevated in MHD patients compared with normal range. Non-survivors demonstrated significantly more elevated levels of CRP (151.90 [115.48, 203.25] verses 64.70 [11.50, 67.90], P=0.011), IL-6 (98.19 [57.78, 151.60] verses 14.62 [8.86, 43.61], P=0.037) and procalcitonin (3.04 [2.36, 3.50] verses 0.80 [0.67, 1.18], P=0.009) compared to survivors, indicating dysregulation of immune response might be highly involved in the pathological process of COVID-19, contributing to the poor prognosis.

### Dynamic trajectories of major laboratory markers of survivors and non-survivors during hospitalization

**Figure 2:**
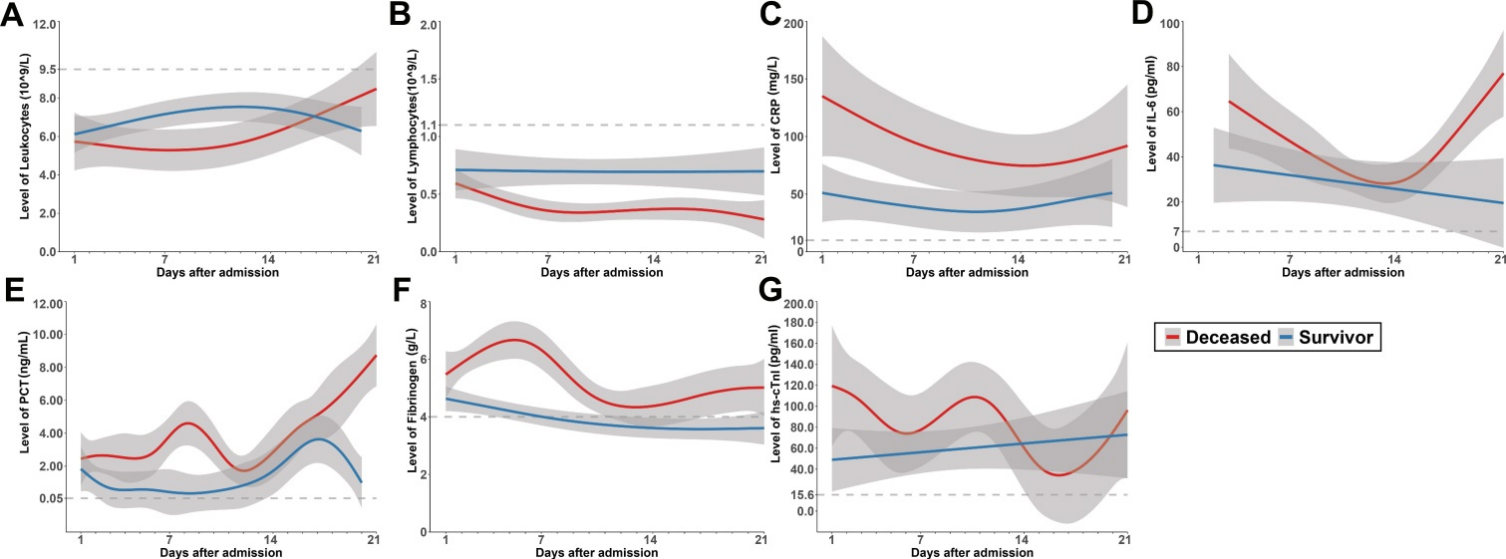
Dynamics of leukocyte count (A), lymphocyte count (B), CRP (C), IL6 (D), Procalcitonin (E), fibrinogen (F) and hs-cTnI (G) in survivors (blue) and deceased group(red) during hospitalization, with 95% confidence interval represented by shaded regions. The dotted line represents the normal value.

Dynamic trajectories of leukocyte count, lymphocyte count, CRP, IL6, procalcitonin, and fibrinogen during hospitalization were tracked using a generalized addictive model (Figure 2). In survivors, the leukocyte level was mildly higher than non-survivors at early phase during hospitalization, it rose gradually in non-survivors, both groups stayed in the normal range during hospitalization. Lymphocyte count was significantly lower in non-survivors compared with survivors during hospitalization, and it continued declining in non-survivors, both groups were below the normal range. Through hospitalization, CRP, IL6 and procalcitonin levels at various points were significantly higher among patients who experienced unfavorable outcome. Although CRP was gradually declining in non-survivors, it was higher compared with that of survivors throughout observation period from admission. IL6 showed a sharp rise in deceased group along with illness deterioration. Similar with IL6, procalcitonin showed a sharp rise in deceased group as well. Fibrinogen had a dramatic rise at early phase, presumably as an acute phase response. However, a sudden decrease was observed shortly before death in non-survivors. Cardiac injury marker hs-cTnI level was above normal range in both groups, it was more elevated in non-survivors and had a fluctuation during the clinical course.

### Lymphocyte subset analysis in MHD patients with COVID-19

Lymphocyte subsets were analyzed in 8 MHD patients with COVID-19 on admission, all of these 8 patients survived and discharged during the follow up (Supporting Table 1). The total number of lymphocytes including B cells, T cells and natural killer (NK) cells were significantly decreased. T cells were shown to be more affected by SARS-CoV-2 as T cell count was nearly half the lower reference limit (469.88/uL). The subsets of T cells were further analyzed. The mean number of both helper T cells/Th cells (CD3+CD4+) and suppressor T cells/Ts cells (CD3+CD8+) were all nearly half the lower range.

### Treatment and outcome of MHD patients with COVID-19

The treatment of patients was shown in Table 1. 78.9% of MHD patients were given antiviral therapy, 47.3% underwent antiviral therapy, 57.8% of the patients took traditional Chinese medicine, 89.4% were given oxygen inhalation, 63.2% underwent Continuous renal replacement therapy, the median duration of CRRT is 18.5 [16.75, 30.0] hour, However, no differences had been found between survivors and non-survivors about need for antibiotics, antiviral treatment, noninvasive ventilation, continuous renal replacement therapy (CRRT) and invasive ventilation.

## Discussion

A total of 19 MHD patients with COVID-19 were enrolled in the study. We reviewed here the clinical features of the 19 hospitalized MHD patients (10 male and 9 female). Notably, according to our findings, the inflammatory markers CRP, IL6 and procalcitonin levels at various points were significantly higher among patients who experienced unfavorable outcome. Significantly elevated fibrinogen level were also observed in non-survivors than survivors, suggesting that uncontrolled inflammation and coagulation abnormalities increased the risk of mortality in MHD patients.

At presentation, MHD patients exhibited similar symptoms (fever, cough, short of breath) as general population, diarrhea seemed more common in non-survivors. It has been reported that dialysis patients have an extended period of viral shedding after SARS infection 6, but we do not observe longer virus shedding duration of COVID-19 in MHD patients. The median duration of viral shedding was 20 days [IQR 15.0, 35.0] for survivors, which was the same compared to survivors in general population (20.0 days [17.0-24.0], n=131) 7.

MHD patients with COVID-19 had elevated infection-related biomarkers, including CRP, ESR, procalcitonin, IL-6, IL-2R and TNF-α, which likely reflected an exacerbated inflammatory response. Although dialysis patients have impaired immune defense 8, they are able to mount immunological response to the coronavirus. Cytokines may play an important role in the immunopathology of COVID-19 9. In our study, MHD patients experiencing an unfavorable outcome showed significant increasing CRP, IL6 and procalcitonin levels compared to survivors. Available data suggests CRP is often higher in COVID-19 patients with worse outcomes 10, which is also observed in our study. Studies have shown IL-6 and procalcitonin were associated with COVID-19 related in-hospital death 7, 11. IL-6 and CRP have emerged as the best predictors of all-cause mortality in dialysis patients in several cohort studies 12-14. Based on these evidences, CRP, IL6 and procalcitonin levels may serve as potential markers for predicting progression of MHD patients with COVID-19.

Further more, our result showed that 94.7% (n=18) MHD patients with COVID-19 had elevated levels of D-dimer. A study of 1099 patients with COVID-19, elevated D-dimer was found in 46% patients^15^. Thus coagulation abnormalities had higher incident rate in MHD patients with COVID-19. In our study, elevated D-dimer, fibrinogen and LDH were more likely to be seen in patients with non-surviving outcomes.

Our study has some limitations. First, though MHD patients with COVID-19 was not commonly seen, interpretation of our findings might be limited by the small sample size. Second, some laboratory tests (for example, lymphocyte subsets) were unavailable in emergency circumstances, and missing data or important tests might lead to bias of clinical characteristics. Therefore, further study is warranted to gain a better understanding of risk factors and outcome of MHD patients with COVID-19, which ultimately may help to guide efforts aimed at reducing the mortality.

In conclusion, MHD patients with COVID-19 who experienced unfavorable outcome had more elevated CRP, IL6 and fibrinogen as well as procalcitonin levels at various points compared to survivors. Our study suggested that the dysregulation of immune response as well as coagulation might be highly involved in the pathological process of COVID-19, contributing to the poor prognosis in MHD patients.

## Figures and Tables

**Table 1:** Demographics and clinical characteristics of MHD patients enrolled in this study

Parameters	ESRD patients (N=19)	In-hospital death (N=6)	Survivors (N=13)	*P* value
Age-yr, mean (SD)	63.11 (11.81)	63.83 (10.57)	62.77 (12.74)	0.861
Range	40-81	50-79	40-81	-
Age ≥65, n (%)	10 (52.6)	2 (33.3)	8 (61.5)	0.350
Male, n (%)	10 (52.6)	3 (50.0)	7 (53.8)	0.628
KT/V, mean (SD)	1.24 (0.20)	1.87 (0.47)	1.25 ( 0.22)	0.541
Dry body weight, mean (SD)	64.2 (3.1)	67.9 ( 4.2)	61.1 ( 4.3)	0.293
Dialysis-yr, median (IQR)	3.0 [1.0, 4.0]	2.65 [1.85, 4.5]	3.0 [0.5, 3.5]	0.198
Days from illness onset to death, median (IQR)	-	21 [7, 28]	-	-
Days from illness onset to discharge, median (IQR)	-	-	22.5 [17.5, 40]	-
Virus shedding-days, median (IQR)	-	-	20 [15, 35]	-
**Primary diseases of ESRD, n (%)**				
Diabetic nephropathy	7 (36.8)	2 (33.3)	5 (38.5)	0.378
Hypertensive kidney disease	6 (31.6)	2 (33.3)	4 (30.7)	0.652
GN	6 (31.6)	2 (33.3)	4 (30.7)	0.652
**Previous hemodialysis access, n (%)**				
AVF	18 (94.7)	6 (100.0)	12 (92.3)	-
CVC	1 (5.2)	0	1 (7.6)	-
**Any comorbidity**				
Hypertension, n (%)	15 (78.9)	6 (100.0)	9 (69.2)	0.255
Cardiovascular disease, n (%)	6 (31.5)	2 (33.3)	4 (30.7)	0.652
Diabetes, n (%)	2 (10.5)	0	2 (15.3)	0.088
COPD, n (%)	2 (10.5)	1 (16.7)	1 (7.6)	0.456
**Signs and symptoms**				
Fever, n (%)	17 (89.5)	5 (83.3)	12 (92.3)	0.544
Cough, n (%)	17 (89.5)	5 (83.3)	12 (92.3)	0.544
Shortness of breath, n (%)	7 (36.8)	3 (50.0)	4 (30.7)	0.617
Diarrhea, n (%)	4 (21.1)	3 (50.0)	1 (7.6)	0.071
Severe pneumonia, n (%)	6 (31.5)	4 (66.6)	2 (15.4)	0.046
SOFA score	6.0 (3.5-8.0)	6.0 (3.5-8.0)	6.0 (4.8-7.0)	0.897
**Treatment**				
Antiviral therapy, n (%)	15 (78.9)	6 (100.0)	9 (69.2)	0.255
Antibiotic therapy, n (%)	9 (47.3)	5 (83.3)	4 (30.7)	0.057
Traditional Chinese medicine, n (%)	11 (57.8)	3 (50.0)	8 (61.5)	0.506
Oxygen inhalation, n (%)	17 (89.4)	6 (100.0)	11 (84.6)	1.000
Noninvasive ventilation, n (%)	5 (26.3)	3 (50.0)	2 (15.4)	0.262
Invasive ventilation, n (%)	1 (5)	1 (16.7)	0	0.315
Continuous renal replacement therapy (CRRT), n (%)	12 (63.2)	4 (66.6)	8 (61.5)	0.621
Duration of CRRT(hour), median (IQR)	18.5 [16.75, 30.0]	18.5 [16.75, 34.0]	19.25 [13.5, 30.0]	0.209

Data are presented as mean (SD) or medians (IQR), n (%).

**Table 2:** Laboratory findings in MHD Patients with COVID-19 at admission

Parameters	Normal, range	All patients (N=19)	Non-survivors (N=6)	Survivors (N=13)	*P* value
Leukocyte count, ×10^9^/L	3.5-9.5	5.91 [4.35, 6.66]	4.63 [4.33, 6.05]	6.33 [5.20, 6.80]	0.349
Neutrophil count, ×10^9^/L	1.8-6.3	4.38 [3.27, 5.53]	3.87 [3.27, 5.36]	4.65 [3.42, 5.24]	0.708
Lymphocyte count, ×10^9^/L	1.1-3.2	0.72 [0.51, 1.10]	0.58 [0.51, 0.66]	1.00 [0.66, 1.12]	0.061
Lymphopenia, n (%)	-	16(84.2)	6(100)	10(76.9)	0.517
Platelet count, ×10^9^/L	125-350	171.50 [114.75, 199.00]	145.00 [115.50, 178.25]	180.50 [115.75, 268.25]	0.426
Hemoglobin, g/L	115-150	92.50 [73.00, 101.50]	93.00 [78.00, 96.75]	88.50 [72.50, 103.00]	0.963
Anemia, n (%)	-	17(89.4)	5(83.3)	12(92.3)	0.544
Creatinine, umol/L	45-84	852.00 [749.00,1115.50]	1063.50 [894.75,1301.25]	776.00 [726.00, 991.00]	0.044
Blood urea nitrogen, mmol/L	1.7-8.3	28.10 [18.60, 37.65]	32.05 [28.25, 39.90]	26.93 [16.70, 36.60]	0.219
Sodium, mmol/L	136-145	137.90 [135.00, 139.35]	136.50 [134.38, 141.10]	137.90 [136.00, 138.50]	0.861
Potassium, mmol/L,	3.50-5.10	5.05 [4.52, 5.68]	5.51 [5.17, 5.89]	4.97 [4.44, 5.40]	0.114
Calcium, mmol/L	2.20-2.55	2.10 [1.98, 2.24]	2.17 [2.10, 2.25]	2.08 [1.96, 2.23]	0.272
Phosphorus,mmol/L	2.20-2.55	1.77 [1.61, 1.87]	1.74 [1.68, 1.78]	1.79 [1.38, 1.87]	0.671
Creatine kinase, U/L	18.0-198.0	63.00 [25.00, 152.50]	125.50 [69.25, 200.00]	32.00 [22.00, 120.00]	0.295
CK-MB, ng/mL	≤6.3	219.35 [152.27, 598.43]	592.85 [307.53, 925.83]	199.60 [128.03, 247.22]	0.137
hs-cTnI, pg/mL	≤15.6	40.65 [12.68, 129.75]	111.20 [59.88, 264.50]	30.55 [10.33, 95.05]	0.275
NT-proBNP, μg/mL	≤486	19651.00 [10727.00, 34480.00]	25785.00 [17823.00, 37455.00]	17543.00 [10727.00, 34480.00]	0.733
Cardiac injury, n (%)	-	15(78.9)	6(100)	9(69.2)	0.255
Alanine aminotransferase, U/L	≤41	11.00 [7.50, 15.00]	12.00 [11.00, 15.25]	9.00 [7.00, 15.00]	0.333
Aspartate aminotransferase, U/L	≤40	19.00 [14.00, 22.50]	25.00 [18.25, 32.50]	15.00 [13.00, 20.00]	0.113
Albumin, g/L	35.0-52.0	32.60 [31.45, 38.15]	32.55 [29.05, 34.55]	32.60 [31.60, 38.30]	0.292
Lactate dehydrogenase, U/L	232-410	276.00 [208.50, 367.00]	439.00 [301.00, 594.25]	233.00 [208.00, 283.00]	0.054
Fibrinogen, g/L	2.00-4.00	5.17 [4.53, 5.69]	6.16 [5.72, 6.62]	4.81 [4.18, 5.19]	0.006
>4, n (%)	-	17(89.4)	6(100)	11(84.6)	0.832
D-dimer, ug/ml	≤0.5	2.03 [1.20, 4.28]	2.07 [1.64, 2.83]	2.03 [0.93, 5.04]	0.599
>0.5, n (%)	-	18(94.7)	6(100)	12(92.3)	0.684

Data are presented as medians [IQR], n (%).

**Table 3:** Infection-related biomarkers in MHD patients with COVID-19 at admission

Parameters	Normal, range	All patients (N=19)	Non-survivors (N=6)	Survivors (N=13)	*P* value
hs-C-reactive protein, mg/L	<10	67.20 [29.80, 114.00]	151.90 [115.48, 203.25]	64.70 [11.50, 67.90]	0.011
Erythrocyte sedimentation rate, mm/h	<15	39.00 [31.00, 75.00]	92.00 [62.00, 96.00]	36.00 [28.50, 47.50]	0.079
Interleukin -6, pg/ml	<7.0	39.67 [11.09, 60.91]	98.19 [57.78, 151.60]	14.62 [8.86, 43.61]	0.037
Interleukin-1β, pg/ml	<5.0	4.90 [4.90, 4.90]	4.90 [4.90, 4.90]	4.90 [4.90, 4.90]	0.546
Interleukin-2R, U/mL	223-710	2091.00 [1354.50, 2564.00]	2162.50 [1636.75, 2568.00]	2091.00 [1306.50, 2546.50]	0.602
Interleukin -8, pg/ml	<62	17.60 [10.70, 22.35]	18.15 [15.75, 20.17]	17.00 [10.70, 22.35]	0.602
Interleukin -10, pg/ml	<9.1	4.90 [4.90, 7.40]	4.90 [4.90, 5.25]	5.80 [4.90, 13.75]	0.257
Tumor necrosis factor-α, pg/ml	<8.1	25.10 [17.80, 28.40]	24.70 [19.80, 29.57]	25.10 [17.75, 27.70]	0.361
Procalcitonin, ng/ml	0.02-0.05	1.31 [0.74, 2.68]	3.04 [2.36, 3.50]	0.80 [0.67, 1.18]	0.009

Data are presented as medians [IQR].
